# It Is “All About Relationships” in Lifestyle Programmes for Adults Living With Type Two Diabetes Underpinned by a Person/Whānau-Centred Care Approach

**DOI:** 10.3389/fresc.2022.829542

**Published:** 2022-04-29

**Authors:** Leigh Hale, Christopher Higgs, Donna Keen, Catherine Smith

**Affiliations:** Centre for Health, Activity, and Rehabilitation Research, School of Physiotherapy, University of Otago, Dunedin, New Zealand

**Keywords:** type II diabetes, exercise, education, self-management, self-efficacy, person centred care, adults, rehabilitation

## Abstract

**Background:**

Lifestyle programmes are important in the management of type 2 diabetes (T2D). The Diabetes Community Exercise Programme (DCEP) is an exercise and educational programme for adults living with T2D with the aim of enhancing exercise self-efficacy and supporting wellbeing. DCEP is underpinned by a model of person/whānau-centred care and the spirit of Motivational Interviewing. Person-centred care models in the context of rehabilitation and long-term health conditions are still evolving. This paper explores what those involved in DCEP perceived important to its person/whānau-centredness.

**Method:**

An evaluative qualitative methodological approach was used with data collected by open-ended interviews and a focus-group at completion of the initial 12-week part of DCEP. Interviews were audio-recorded and transcribed verbatim. Participants were 16 DCEP attendees and 13 healthcare professionals (HCPs) involved in DCEP delivering. Data were analysed using the General Inductive Approach.

**Results:**

The three interconnected themes constructed from the analysis were ‘*It's all about relationships’, Balancing the Outside World*, and *Empowering through Knowledge*. Through the experience and motivation of positive person-centred relationships, DCEP addressed attendees' T2D health needs, but they were constantly balancing these needs with those of family, employers, finances, other health needs and life interests. DCEP relationships facilitated ongoing discussions between attendees and between attendees and HCPs. The ability to discuss in a supportive and non-judgmental environment helped attendees to make sense and process the information they gained at DCEP. This empowering through knowledge in turn helped attendees to work out ways of balancing the outside world and thus better address their T2D needs.

**Conclusion:**

It is “all about relationships” was key to DCEP's person/whānau-centred approach—all other factors related back to the development and maintenance of relationships. These relationships were between all whānau involved: the attendees, their family, the wider community, the healthcare provider organisations, and the HCPs and personnel involved in delivering DCEPs. These relationships created an accepting, understanding and social atmosphere that enabled attendance and facilitated both knowledge exchange and ability to exercise, both considered to be beneficial by attendees. Importantly, these relationships took time to develop, but the benefits were worth the investment.

## Introduction

As with most countries world-wide, type 2 diabetes (T2D) is considered a serious health challenge for Aotearoa New Zealand (NZ). The NZ Ministry of Health have made it a priority long-term condition and have developed a holistic management plan, Living Well with Diabetes, to improve the outcomes for people living with T2D (1). This plan's vision is that “all New Zealanders with diabetes, or at high risk of developing type 2 diabetes, are living well and have access to high-quality, people-centred health services.” The plan is underpinned by quality standards. One key quality standard is “All people with diabetes will receive personalised expert advice on lifestyle choices such as good nutrition and regular physical activity together with help with behaviour change, smoking cessation advice and support if required” (2). Recommendations for personalised education, for both lifestyle factors and medication use, have been recently highlighted as ways of addressing the barriers to T2D management contributing to the ethnic health inequities experienced in NZ (3, 4).

Participation in exercise and/or physical activity is important in the management of T2D (5), but given the long-term nature of T2D, it should be lifelong engagement, and this can be challenging to achieve (6). Sociological factors, such as sense or absence of wellbeing and feelings of safety among others, vulnerability to failure, aversion and shame can influence why and how a person engages in exercise or physical activity (7). To our knowledge, no study has reported on the physical activity levels of people with T2D in NZ. What is known however, is that there are disproportionally more Māori and Pacific people with T2D than in other ethnic groups and that one way to address these inequitable health outcomes is to provide culturally appropriate and accessible non-pharmaceutical lifestyle interventions, such as physical activity programmes (8, 9). Few studies have reported on physical activity engagement in Indigenous peoples in NZ and Australia and thus it is unclear what type of interventions would be successful in achieving this in these communities (8). Several lifestyle programmes specifically for Māori and Pacific people living with T2D have been developed in NZ and found to be acceptable and potentially beneficial (9–15). Sustaining these programmes is often challenging, contingent on funding models (10). A lifestyle programme developed in our region of the South Island of NZ in 2003, although found to be acceptable, beneficial and engaging, was only able to access further central government health funding to continue for 1 year and has since been discontinued (10).

In response to this gap in health services in our region of NZ, in 2008 we developed the Diabetes Community Exercise Programme (DCEP) to encourage people living with T2D and multimorbidity to engage in exercise (16). Engagement and self-efficacy to exercise and live well was facilitated by participant-driven education. In the evolving development of this programme we endeavoured to ensure cultural appropriateness and accessibility for Māori and Pacific people, as well as holding true to the Ministry of Health's directive that services should be people-centred (2).

Put simply person-centred care involves an equal partnership between the care recipient and the healthcare professionals; collaborative healthcare that meets the needs, values, and desired outcomes of the care recipient (17).The nature and role of person-centred care in the management of long-term health conditions is however complex, and in the context of rehabilitation and long-term health conditions, the concept is still evolving (18–20). In nursing care, the concept is more evolved. One model of person-centred care described in nursing comprises four constructs (i) prerequisites, which focus on the attributes of the healthcare professional; (ii) the care environment, which focuses on the context in which care is delivered; (iii) person-centred processes, which focus on delivering care through a range of activities; and (iv) expected outcomes, which are the results of effective person-centred care (21). McCormack conceptualised the meaning of “person” in person-centred care as being: in relation, in a social world, in place, and with self (22).

To facilitate engagement of Māori and Pacific people we were guided by our obligations to the NZ's Treaty of Waitangi, mindful of the principles of partnership and participation, and the Ministry of Health's Māori and Pacific Health Action Plans (23, 24). To this end we consulted Māori and Pacific communities and worked collaboratively with a Māori long term conditions nurse and the local Pacific Trust. To ensure a people-centred focus we adopted the emerging NZ model of a whānau (family)-centred model of care, described as “a culturally grounded, holistic approach focused on improving the wellbeing of whānau as a group, as well as the individuals within the whānau” [25, p. 17]. The concept of whānau in NZ extends in complexity beyond the traditional concept of family to broader collectives and has physical, emotional, spiritual, and genealogical dimensions; it is thus multi-layered, flexible and dynamic (26). As described in Physiotherapy NZ's Person- and Whānau-centred Model of Care “Whānau is a concept that applies to many cultures and ethnicities. Whānau are those to whom the person relates in terms of shared experiences, values and beliefs. The people and relationships that comprise a person's whānau may be lifelong, or time-limited and specific to the person's life circumstances. These relationships are not necessarily reliant on kinship ties” [27, p. 6]. Thus, DCEP was developed to enable attendance of both the individual and whānau as desired, and as the programme evolved, all those involved (attendees and healthcare professionals as well as those in wider the community) became encompassed as whānau. To further enable partnership, the exercise prescription and choice of exercise mode were individually tailored, the education topics were attendee driven and were interactive, and, to facilitate participation, a supportive atmosphere was pursued. Additionally, to assist attendance of those living in lower socioeconomic conditions to attend, the programme was free to attend. At the healthcare professional level, to enable partnership and participation, services were delivered underpinned by the spirit of Motivational Interviewing (MI) (28, 29). Motivational Interviewing is a collaborative communication style designed to explore and strengthen a person's own motivation for and commitment to change within an atmosphere of acceptance and compassion (28). The central tenet of the spirit of motivational interviewing “is an attempt to have a quiet and constructive discussion about change in which the client drives the process as much as possible” [29, p. 112] and is considered the intersection of the four components of MI, namely collaboration, evocation, acceptance and compassion (27). The therapist when using MI is thus a guide rather than an expert.

DCEP has been described in previous publications, but in brief, it comprises an initial twice a week 12-week programme of individually tailored exercise and education sessions, facilitated by a physiotherapist and a nurse, held in a group community setting and followed up by a twice weekly ongoing maintenance exercise class (30).

We previously reported on a qualitative exploration, post the initial 12-week programme, of what DCEP attendees and healthcare professionals (HCPs) who delivered the programme perceived DCEP to be and what motivated attendance. People did engage in DCEP and the key factors identified that encouraged participation were its person-centred focus and social atmosphere. Knowledgeable healthcare professionals trained in delivering person-centered care and well-trained administrative support to organise the programme were also considered important (31, 32). These findings were encouraging as they reflected the person-/whānau-centred approach we were seeking.

Considering the knowledge that a focus on person-centered care appeared to enable engagement in DCEP, this paper deepens our previous qualitative exploration to understand what DCEP attendees and the HCPs delivering the programme considered to be key “constituents” of DCEP and whether these constituents reflected the person/whānau-centred approach we aspired to. A thorough understanding of these constituents would not only assist in refining DCEP but would inform similar programmes aimed at improving health and wellbeing of people living with long-term conditions and multimorbidity as well as add to the evolving understanding of person-centred care in rehabilitation. Thus, this study addressed the following research question: what were aspects of DCEP considered by attendees and HCPs to be important to its person/whānau-centredness?

## Methods

### Design

To address our research question, and in keeping with our aforementioned study, qualitative evaluative methodology (33) was applied, using semi-structured interview guides in individual interviews or focus groups to collect data and the General Inductive Approach to analyse it (34). The General Inductive Approach is described as a systematic analysis which is guided by the specific research objectives. One of the underlying assumptions is that the analysis is both deductive (driven by the research objectives) and inductive in that the findings are developed from multiple readings and interpretations of the raw data (inductive). So, the analyst castes a specific lens to the raw data, in this case what were participants saying that reflected a person/whānau-centredness. The interviews formed part of a process evaluation embedded within a randomised controlled trial (RCT) investigating the effectiveness of DCEP compared to usual care. In the current study we analysed the data from these interviews specifically related to our research question. A further assumption of the General Inductive Approach is that, due to its deductive–inductive nature, the findings are influenced by the assumptions and experiences of the research team. Thus, knowing who the research team are is important to the understanding of the presented findings.

The research team comprised three females and one male [three academic physiotherapists (two with Doctoral degrees, one with a Master's degree) and one academic sociologist (with a Master's degree)]. All had extensive experience in qualitative research and held a constructionist stance. Additionally, all had research experience (and three with clinical experience) working with Māori and Pacific peoples and with those living in low social economic background. However, all were Pākehā (non-Māori), and thus were limited in their ability to cast a true cultural perspective to the research.

The RCT is registered with the Australian New Zealand Clinical Trials Registry (ANZCTR): ACTRN12617001624370p, the protocol of which has been described previously (30). The NZ Health and Disability Ethics Committee (17/CEN/241) approved the study. This report adheres to the COREQ (COnsolidated critia for REporting Qualitative research) checklist (35).

### Recruitment and Eligibility

Recruitment into the RCT was *via* health care providers or public media advertising. Participants for the interviews were recruited from those randomised to the intervention arm of the RCT (attendees) and from all health care providers (HCPs) involved in the delivery of DCEP. Attendees were adults (>35 years) diagnosed with T2D who had clearance from their general practitioner (GP) to participate in exercise. During the consenting process for the RCT, attendees also indicated consent, or not, to be recruited into the process evaluation study. The RCT was conducted in two southern NZ cities and recruitment was over 7 waves (3 waves in city 1 and 4 waves in city 2) with a total of 83 participants randomised to the DCEP group. On completion of the 12-week DCEP programme 2 to 3 consenting DCEP attendees from each wave were sampled for interview, balancing for level of DCEP attendance. HCPs received information pertaining to the process evaluation study and volunteered and consented to participation.

### Data Collection

Data for the current qualitative inquiry came from semi-structured interviews and a focus group conducted immediately post the 12-week DCEP intervention. Whilst the qualitative interview guide, developed by the research team, was based on the Consolidated Framework for Implementation Research (CFIR) (36), the richness and depth of the collected data enabled a deep analysis of DCEP itself. The included questions explored attendees and HCPs experience of DCEP, what could have been done better or differently, how well it met the needs of attendees, how it influenced motivation to exercise/clinical practice, and what they thought were the important components of DCEP. Data were collected by experienced qualitative interviewers known to participants. The interviewers were all female and from diverse ethnic [Māori, Pākehā (non-Māori)] and academic (nursing, psychology, social science) backgrounds. DCEP attendees were interviewed independently or with support from family/whānau at a mutually agreeable venue (homes or clinic). The HCPs were either interviewed independently (*n* = 8) or *via* a focus group (*n* = 5), depending on their preference or for logistical reasons, at a clinical venue. The interviews took in duration between 16 min and 1 h and the focus group took 1 h, following which the interviewers wrote field notes for use in the data analysis. All interviews and the focus group were audio-recorded. A commercial transcribing firm transcribed word-for-word all interviews. There were no follow-up interviews or member checking undertaken.

### Data Analysis

The General Inductive Approach guided analysis (34). In keeping with the principles of a whānau-centred approach, where whānau is viewed as a wider collective of people with inter-related connections, the data analysis combined the views of the HCPs and the attendees and each group was not analysed separately. In this process the audio recordings were first listened to and then the transcripts were read multiple times by two researchers (CS, LH) during which time notes were made of text that related to addressing the research question, and preliminary codes began to be developed. These notes and early codes were discussed and compared alongside the field notes. Further discussions resulted in a lower order coding scheme which then one researcher (CS) applied across all transcripts. As more transcripts were coded, higher order codes began to be identified and were placed in an excel spreadsheet. In sequential researcher meetings, the wider team (CS, LH, CH, DK) discussed and reflected on these higher order categories and research themes were constructed. Coded text illustrating these themes were identified and transferred to the spreadsheet to sit alongside the theme. As the themes took shape, they were further discussed and agreed upon. In the analysis of the final transcripts no new codes or themes were found signifying data saturation.

### Trustworthiness

In addition to sharing and seeking advice on the advancing analysis and theme development with the wider research team members, the final analysis was presented, discussed and verified with DCEP attendees and HCPs in workshops held in both cities, a method recommended in the General Inductive Approach (28).

## Findings

Sixteen DCEP attendees (11 females, aged 39–76 years) (Table 1) and 13 (11 females) health care professional participants (HCPs) (physiotherapists = 2, nurses = 4, programme developers = 2, pharmacist = 1, podiatrist = 1, dietitian = 1, administrator = 1, counsellor = 1) consented to participate in this qualitative evaluation, and 8 were independently interviewed and the remaining 5 were interviewed in one focus group.

**Table 1:** DCEP attendees age, sex and ethnicity data.

**Participant**	**Age (years)**	**Sex**	Ethnicity^*^
P175	53	F	NZE
P205	72	M	NZE
P238	41	F	NZE
P280	70	F	NZE
P324	63	F	NZE
P373	70	M	NZE
P519	55	F	NZE
P529	73	F	Cook Island Māori
P542	73	M	Māori
P595	76	M	NZE
P639	54	F	Māori
P686	54	F	NZE
P887	51	F	NZE/Māori
P917	72	M	NZE
P920	56	F	NZE
P933	64	F	Māori

*DCEP, Diabetes Community Exercise Programme; NZE, New Zealand European*.

* *As per New Zealand Census (New Zealand European, Māori, Samoan, Cook Island Māori, Tongan, Niuean, Chinese, India, Other) each ethnic group includes all those who have identified with it, so people may be counted in more than one group*.

Our analysis identified three interconnected themes: “**It's all about relationships”**, **Balancing the Outside World**, and **Empowering through Knowledge**. Through the experience and motivation of positive person-centred relationships, attendees felt that DCEP addressed their T2D health needs. However, they found they were constantly balancing their T2D needs with those of whānau, employers, finances, other health needs and life interests. DCEP relationships facilitated ongoing discussions between attendees, and, between attendees and HCPs. The ability to discuss in a supportive and non-judgmental environment helped attendees to make sense and process the information they gained at DCEP. This empowering through knowledge in a whānau-centred way helped attendees to work out ways of balancing the outside world and thus better address their T2D needs.

The themes are described below, illustrated with participant quotes. Quotes attributed to DCEP attendees have a unique numerical number e.g., P238 and HCP quotes are attributed with their profession.

### Theme 1: “It's All About Relationships”

This theme's name came from a quote from DCEP Developer 2—“It's all about relationships”—that encapsulates DCEP. Once attendees started the programme, developing relationships appeared to be a crucial “glue” that strengthened commitment from all involved to DCEP. These relationships, both between and within attendees and the HCPs, are deconstructed within the sub themes “***No mirrors, no lycra, no judgement”*** and ***Camaraderie***.

#### “No Mirrors, No Lycra, No Judgement”

Anxiety was experiencing by several attendees about attending a group programme, a concern also echoed by HCPs: “*That's going to be scary. And, certainly, … I've got memories in the past of some people with significant issues* [social anxiety] *and they've completely frozen. And not come back”* (DCEP Developer 1). But, overtime, this anxiety eased as people began to feel more comfortable with each other: “*I am an awkward bugger … I don't mix with people very well. It takes me a long time to know people and once I know people, I am comfortable within that group… you start talking more freely amongst yourselves”* (P373).

Growing comfortable was facilitated by feeling both welcome and safe, and not judged. A core value of DCEP was acceptance, described by DCEP Developer 1 as “*no mirrors, no lycra, no judgement*”. Further, attendees appreciated how the HCPs would relate and be responsive to both group and individual needs: “*When I was gone for 2 or 3 weeks, there were certainly some, “good to see you back” and what not. Which is nice 'cause you like to think you're missed occasionally*“ (P175). Consequently, attendees developed high regard for the HCP team who they did not want to let down. Attendees noticed absence: “*I hate to see [physiotherapist] out there putting all the equipment out and nobody turning up*” (P373).

Not being judged was particularly valued by attendees: ”*We can go there how we are, we're taken how we are, and get on with it…no one's there to look at what you're wearing or*… “(P933) and made them feel comfortable to contribute to group discussions because: “*Whatever anybody said, it wasn't wrong. Everybody accepted their opinion, your opinion, and, and maybe just offered different ways to, to do something differently.”* (P238).

Staff were described as friendly, approachable, encouraging, with a style perceived as non-threatening: “*It takes a special type of person to lead it and I think* [DCEP physiotherapist and Nurse] *were wonderful. … They have got a fantastic approach and way with people. They never at any point and time made you feel a lesser person or embarrassed … full of encouragement all the way”* (P205).

HCPs interviewed understood that their role was “*to support, and help, and guide”* (Nurse) in a non-judgemental manner, an approach emphasised in training and orientation. The physiotherapists, particularly, explained how they needed to “*let go”* (Physiotherapist) of a more traditional directive approach: “*I know at times I think I felt I'm a little bit too lenient with that I haven't kinda of you know if they can't attend all the sessions I'm kinda like, ‘That's OK*”’ (Physiotherapist). HCPs also recognised the health benefits of being non-judgemental: “*Not judging definitely helps with the relationship because then they trust you a little bit more and sometimes what they tell you is just the tip of the iceberg as well so if you're open to that, then they might just delve a little bit deeper and you can actually start to get down to the real problem”* (Physiotherapist).

In turn, the HCPs experienced positive feelings about adopting a supportive and non-judgemental approach: “*But those who have really got involved and started to have conversations, and meet and build the relationships with patients, I think, see the huge value. It's just nursing that's got a different hat on”* (Nurse manager).

Whilst HCPs thought that a non-judgemental approach could be (at least partially) taught, some described occasions where they felt that other HCPs were hesitant to adopt this role, possibly inhibiting the group fellowship and reducing motivation to attend: “*I think it might be obvious to participants because I'm feeling really uncomfortable and I'm trying not to but I'm really struggling*.” (Physiotherapist). Also important was a good working relationship between the physiotherapist and nurse: “It's *team dynamics, you definitely need a team that can work together and that's not necessarily something you can just choose*” (Physiotherapist).

A further layer of acceptance included the welcome extended to family, friends and support persons. An exercise buddy was encouraged, and this was very motivating for several attendees: “*But um, my husband is great support he likes coming along as well. So, that was probably a big part for me was that I had someone I was able to talk to um, about the programme*” (P324). This welcome was extended to the education sessions: “I *said to* [physiotherapist]*, um, on our last day, you know when the next group starts, when the arthritis man comes could I bring my mum along too? She's got real bad arthritis… and she's* [physiotherapist] *like “I don't think that will be a problem.””*(P238).

Attendees described their groups as being diverse in age, gender, ethnicity, nationality, interests, wealth, mode of transport, and length of time being diagnosed with diabetes. This was perceived a unique aspect of DCEP, reflecting acceptance, and something that would not happen elsewhere in the health system: “*So, there was people from all different walks of life there, that would as a general rule would never have come together. Um, so we had the retired* [professional] *who was clearly very wealthy… then you had the middle of the road people, like myself … there was a couple of …[different ethnicities].. ladies that came … So, we all rock up there in our cars, some of them were in really flash cars and I am in my Corolla, and then you have got these ladies that came on the bus”* (P 686).

#### Camaraderie

This sub-theme describes the supportive and encouraging interactions between attendees during exercise, education sessions and, in the community. Through camaraderie, exercising with the group was a motivator for attendance: “*Well you get motivated because you are with a group and the group you know, you quite enjoy going, I mean I enjoy going but I don't enjoy doing the exercises*” (P595). Attendees greeted, laughed, talked, listened, watched and shared food together. Exercise was not easy for many; some felt uneasy about body image and others experienced pain due to health conditions: “*I also have um, some pretty bad knee pain and I have um, bursitis in my right hip, so I am in pain a fair bit of the time. So, just getting up and down out of a chair or walking from here to the car is you know pretty crap.”* (P686).

C*amaraderie* during exercise appeared to act both as a distraction and as a motivator: “*I mean we back chat and chat and that helps you with your exercises, because he will say to me, what are you standing there for and I will say to him, go on bark a bit more”* (P595).

*Camaraderie* was also experienced in education sessions. Attendees listened to each other, shared experiences and asked questions “*We were in a particularly good group because they asked questions and they were all, as I say the majority were ladies, ah, and they asked some really good sensible in-depth questions, searching questions I would say”* (P205). Attendees actively supported and encouraged each other: “*A couple of them were really good at um including her in conversations that you know she was a bit shy*” (Nurse).

Attendees observed each other's progress and, as well as supporting each other within each session, they also supported each other across sessions. People remembered each other's stories and life events which led to attendees feeling visible and heard, for example: “*And then, one of the guys that on when I went to the maintenance, he was asking me about have you got your date for surgery and I said no. And he just turned around to me and said ‘Oh well, you're doing so well’“* (P238).

HCPs also observed and were rewarded by the *Camaraderie* of the group; however, acknowledged that each group was unique and, some groups were more supportive than others. Some HCPs felt able to facilitate *Camaraderie* intuitively: “*when the bikes are together and things like that, you end up having a conversation with two people at the same time and they end up talking and you kind of move on”* (Physiotherapist). HCPs felt that including more advanced communication skills could help HCP facilitate more positive group dynamics: “*And it's also an endorsing thing, as well, because, actually, sometimes, you need a few more resources in a group to actually shut down conversation that's going to be difficult*” (Nurse).

### Theme 2: Balancing the Outside World

DCEP could not however be seen in a vacuum. Attendees volunteered for the study because addressing their T2D needs was important to them: however, this need was balanced with other challenges, demands and priorities: ”*Admittedly, I haven't gone as often as I could. But we had a drama-filled March, and then I was way too busy*“ (P175). Attendees worked hard to balance health, whānau/family and work with programme attendance. Working attendees felt that the timing of sessions was difficult as they did not fall across regular meal breaks. One attendee indicated that attendance at the programme had affected their income: “*I made it work. It was awkward to a financial decrement to me, slightly. But I am not going to go on about that. But I wanted to be in the study so I made sure that I could be there*” (P887). Attendees prioritised caring for family: “*I think the one, or the odd day that I had no work, I was well enough to go, I had my children because my wife was busy*” (P840). Health needs that prevented attendees from attending included pain severity, conflicting hospital appointments and other undisclosed health complications. Occasionally, attendees just did not feel like attending but managed to overcome this because: “*There has times where I've thought, ‘I don't think I'll go in today, I might have a rest,’ but then I think, ‘no, I'd only be letting myself down.”’* (P542).

Other needs included being warm and sheltered—one attendee who caught the bus would not attend if the weather was bad. Apart from family, health, work, weather and motivation, attendees reminded us that having fun in a way not related to T2D needs was important: “*So, um, I missed quite a few…Just other commitments, like and because I had to go to Jimmy Barnes [Australian rock icon] in Christchurch. Somethings are way more important…”* (P686).

HCPs recognised that attendees had to balance other needs with programme attendance: “*I know like at times I think I felt I'm a little bit too lenient with that like I haven't kinda of you know… if they can't attend all the sessions I'm kinda like, ‘That's OK”’* (Physiotherapist). Furthermore, HCPs recognised that if attendees were forced to make decisions around balancing needs, then they might not choose the programme: “*It's almost prioritising them coming to the group versus not coming and knowing how far you can push or not push with that”* (Physiotherapist). This approach was different from usual practice (e.g., patients charged for no-shows), and although difficult for HCPs, was very much observed and appreciated by attendees: “*I mean you can't force anybody to do it and um, the physio and the nurse um, they didn't push anybody. No comments were made um, nobody was egged on or um, you made to feel guilty if you know, you were having a bad day as some people were, you know they had woken up in the morning and for whatever reason they were feeling awful, they would still come, they would still come and they would still get their bloods and blood pressure taken by [nurse] They may not do very much, they might just sit there and have a chat”* (P324).

### Theme 3: Empowering Through Knowledge

Whilst recruitment into the study was essentially *via* general practice and public advertising, it was recommendations by friends, whānau and other health care providers that made many attendees decided to participate. Most volunteered as they had goals, such as weight loss, being fitter, reducing or stopping medication, and wanting to learn more about T2D. For some, strong commitment to DCEP in of itself was important: “*my only goal was to make sure I got there every time, and I've done it*” (P933).

It was clear that attendees took and brought knowledge to and from DCEP, and this could not be controlled by the HCP. For most attendees, knowledge appeared to be more: sustained, retained, empowering, meaningful and, motivating than other knowledge sources such as HCP encounters in traditional settings and educational days. Knowledge extended beyond passive information delivery and included knowledge-sharing with friends, family and the land.

Several attendees described feeling powerless at and around diagnosis and felt confused about medication, exercise and diet: “*Like I got no, nothing, no support, no, I didn't know what the hell was going on or, and I didn't know like what was normal or what was my normal and I felt a bit like um, alone and like, you know misinformed”* (P238). HCPs also observed this: “*Definitely a trend of people being frustrated that they didn't have that access, or they didn't feel listened to or, even people complaining about getting conflicting information from their different providers, where your GP tells you one thing, and your specialist care provider tells you another. And they were just, like, discombobulated of, like, no idea what way's up anymore*” (Physiotherapist).

Other attendees felt that they were given too much information at once—an approach they perceived as unhelpful as they could not process a large amount of information in a short space of time. This attendee described their experiences at a diabetes information day: “*It is just so hammer, hammer. I mean I didn't see anyone that took, no one was enjoying themselves, no one I don't think really learnt much, apparently*.” (P639).

These perceptions were supported by HCPs who observed: “*So, I think there are, maybe, I think, as diabetics, they just get bashed from all sides and, so, they think, ‘oh my God, another one's* [HCP] *coming to bash us sometimes”’* (Podiatrist).

In contrast, attendees felt that DCEP delivered more meaningful knowledge: “*Um, unique about it. It covered everything, everything you needed to know. Yeah. Not just what they wanted you to… Because, we could say to her, well actually we want to know about this, and she would hook someone up like 3-4 weeks down the track and she would have them there”* (P639).

Meaningful knowledge was motivating for attendees: “*Well I am more motivated, I know what to do, you see it is just learning curve for me at the moment. I didn't, I didn't know what to do for the best, literally to aid and abet this diabetes”* (P917).

Some HCPs were concerned about information retention: “*Because I can see the benefit of this programme. Like, the education is really important, and I can see, but how much do they take away when they just listen? Very little”* (Nurse). However, attendees felt that the opportunity to listen, discuss and ask questions over a 12-week period helped them to retain information: “*Because if you just go for one day, you know all those questions, but over 12 weeks there is different things that crop up that you are able to ask. So just on one day you might not think of everything, but with 12 weeks, yeah. And like with having the, like the podiatrists and the dietician and the pharmacists and all those different people, you know, you kind of thought of something and other people asks questions as well, and you think, oh yeah, I had been wondering about that”* (P920).

HCPs observed that the degree of health literacy varied amongst attendees and were worried that DCEP would be more useful for attendees who had more initial knowledge: “*Just acknowledging that some people are not, you know, people are at all different levels with their learning and sometimes you can kind of get people who are kind of you know here [indicating higher] and that's fine but if you've one person down here [indicating lower] that's quite hard to kind of, for them to engage”* (Physiotherapist). However, the education sessions were appreciated by all attendees and all felt they slowly increased their understanding: “*I didn't have any information before I came to this course. And things throughout the time in the three months that you'll be learning, is was like the little light bulbs were going off* ” (P238).

This gradual process of enlightenment was aided by the attendee-driven discussions: “*I think that made it easier and easier because like, like sometimes when you have discussions, whatever anybody said, it wasn't wrong. Everybody accepted their opinion, your opinion, and, and maybe just offered different ways to, to do something differently. Gave you another perspective on, on stuff* ” (P238).

An important aspect for attendees was the opportunity to share knowledge in a network that involved friends, family, community and the land. Being able to bring support persons (friends and family) to session was helpful: “*I think some of the others the wives and spouse's ah they got a lot out of the programme, and I think it gave them an understanding of, of exactly what it is that ah diabetes is and um, and how they can um, be a support. I know* [spouse] *is asking me in the mornings, oh how is your blood sugar this morning, whereas before you know, either of us probably you know*, [were not] *too concerned”* (P324). Furthermore, attendees described experiences where friends and family gained new knowledge through the programme that positively influenced their own health: “*It has planted the seed and each time I say I am going to do more, like hubby he's going to see somebody at Sport Southland…this has motivated him to go there to do something, so he has started to um, go for walks at night”* (P887). In addition to engaging with discussion within the group, attendees also shared diabetes information outside the DCEP programme: “*quite a good friend, her 11-year-old daughter, they discovered she had type 1 diabetes and like she, her mother didn't have a clue of, and so I sort of knew of places that she could go to for help through, yeah having been* [to the DCEP programme]” (P920).

Attendees also wanted to share knowledge because they were concerned about the health of their communities: “*Goes from me to the next one and then next one. Yeah. And um, unfortunately we still need a lot of work in our health area…And making it, like making it the norm. You know, because I mean there is still the way some of us are eating now is still abnormal for our community. You know it is still junk food…”* (P639).

Attendees also talked about nutritional knowledge with regards to the land with regards to the importance of knowing about, sourcing and, eating locally grown food: “*Only other thing that's there for me is about the land. For me food wise it's about what I can if I can get stuff that's worse than so many kilometers of the house as opposed to hundreds of kilometers. That means to me that for me things are fresher and so are more likely to be better for me.”* (P146).

For some attendees, being empowered with knowledge was difficult. Being reminded about the health effects of diabetes was sad for some: “*It made me more aware of my body and yes and the damage I've been doing to it actually. It's made me not want to continue because I don't want to pass early*” (P503). Whilst most attendees were happy with the *acceptance* shown by HCPs, occasionally, an attendee would experience a less helpful approach: “*That dictation type thing you know, dictating to people is, it just doesn't work, well it doesn't wash with me”* (P639). Sometimes they received confusing and conflicting knowledge: “*From the programme and I got really confused, I got really confused about what is right and what is not right. I had started on a diet control management of diabetes and it was working for me and they were telling me something different…”* (P639).

## Discussion

We explored what those involved in DCEP perceived to be important to its person/whānau-centredness and these were: the importance of relationships, the recognition that people must balance attendance around other priorities in their lives, and the gradual development of co-constructed understanding and knowledge formed reciprocally through respect and trust. Whilst people said they initially attended because they had health-related goals, such as weight loss, being fitter, reducing or stopping medication, and wanting to learn more about T2D, it was the person-centred/whānau focus that appeared to encourage continued attendance in the 12-week programme. These findings reflect McCormack's conceptualisation of the “person” in person-centred care (22, 23). That persons exist in relationships with other persons (being in relation), they are social beings (being in social world), they have context through which their personhood is articulated (being in place) and being recognised, respected and trusted as a person impacts on a person's sense of self (being with self) (22). The findings also resonate strongly with those of McKenzie et al. (37), which identified the key mechanisms of engagement (and the importance of relationships), focus (on the person) and empowerment (to enhance self-efficacy to change health behaviour), encouraging nurses [and physiotherapists in this case] to “actually listen” to their patients and not just “fix them” (p. 301). This too aligns with the relational component of MI that is underpinned by a focus on collaboration and empathy in the helping relationship (38) and the role of engagement in MI (28) suggesting that DCEP had elements that were consistent with the spirit of MI.

The developing nature of the multi-directional relationships and co-creation of knowledge during the initial 12-week DCEP programme reflects what Gibson et al. called “care(ful) tinkering” [39, p. 1529] leading to the outcome that Toff et al. described as “importance of wellbeing as a sense of feeling at home in physical activity” [7, p. 11]. Our findings build further understandings of person/whānau-centred care within the rehabilitation context of long-term health conditions. These findings demonstrate the complexities of person-centered care in this context, and the enduring nature of it, in that to develop relationships of power reciprocity and trust that enable vulnerable people to not only build an inner sense of wellbeing, but to flourish, takes time and care. This is not a brief rehabilitation interaction expecting short-term positive outcomes, but a lengthy one with an expectation of enduring positive outcomes.

In T2D management, the key positive outcome is improved glycaemic control (40). In the rehabilitation context, a combination of aerobic and resistance exercise has been proven effective to improve glycaemic control in T2D, but only if undertaken at the dose (intensity and frequency) prescribed (41, 42). As T2D is a long-term condition, it stands to reason that people will have to exercise at this prescription dose lifelong. Clinical trials, and indeed HCPs, will talk about adherence and compliance, in this case to exercise programmes. Such pejorative terms do not consider the “person” as conceptualised by McCormack (22). Many people with T2D, such as those participating in our trial, live disenfranchised lives, for instance with very poor health (multimorbidity, polypharmacy), cultural inequities, low socio-economic, or challenging social conditions. Such marginalisation does not facilitate engagement in exercise (7). Our DCEP attendees did however engage in DCEP as they considered their “person” had been attended to—they spoke of the relationships (being in relation and in a social world), being enabled to engage whilst balancing the outside world (being in place) and being empowered through knowledge, not just for themselves but being able to use this knowledge for others (being with self).

The key finding in this study was the importance of relationships, and how this underpinned engagement, echoing Mc Kenzie et al. findings (37) and those of Allory et al. (43). In the latter study, people with T2D living in a deprived area of France who attended a diabetes self-management education programme spoke of community relationships—the familiarity of environment (being in place) and with educators (being in relation)—as important facilitators in engagement (43). In our study, relationships were multi-directional between HCPs, between attendees, between HCPs and attendees, and between all and the external community, encapsulating the meaning of “whānau”. The crucial role of the HCP in building these relationships was pivotal, and whilst some HCPs were skilled at interpersonal interactions, others had to learn to be supportive as opposed to what Norris and Kilbride (44) termed being “benign dictators” (p. 2).

Empowering through knowledge is an interesting enigma in healthcare. Educating patients appears to be one of the mainstays of long-term condition self-management, indeed one quality standard of the NZ Ministry of Health's Living Well with Diabetes plan is “All people with diabetes will receive personalised expert advice on lifestyle choices …”. The experts will give advice. Ross et al. (45) explored HCPs attitudes and beliefs towards diabetes self-management education and reported the tensions and opposing viewpoints held by HCPs from not believing that people can self-manage their conditions so why refer to such education programmes, to others becoming frustrated with patients not taking responsibility for their own health in spite of attending education programmes. There was little evidence of HCPs spending time in developing the relationships necessary to enhance patient learning. Indeed, the focus on patient responsibility has ignored the complexities of people's lives, where social relevance, appropriateness and priorities impact on what they can achieve, even if the knowledge is gained and understood. This is particularly so for marginalised or disadvantaged groups (46). For registered HCPs in New Zealand, cultural competence is a key requisite. Epner and Baile (47) argue that culture is complex, a “elusive and nebulous” concept, and that rote learning cultural competence relegates it to inauthenticity and that the key to cultural competence lies within the person-centred care approach. In other words, focusing on the person (22) and developing a true relationship. In the current study, being empowered through knowledge was evident in how DCEP attendees spoke of the educational sessions. These sessions were purposely attendee-driven to enable attendees to request the knowledge they felt they needed and to allow this knowledge to develop slowly over time, enhanced by peer interaction and peer learning. Motivational interviewing in groups can help people become more empowered to manage their health whilst also providing valuable social support (48). Hughes et al. (46) suggest that allowing space for peer support and experiential knowledge in group interaction is important and a balance between prioritising what HCPs considered important knowledge and what patients consider relevant needs to be reached. Whilst the HCPs in our study endeavored to enact this, some expressed feeling insecure and anxious working in this space especially when they could not predict what would be asked of them. Training HCPs to feel secure and supportive in this type of sharing environment is necessary.

An interesting finding of our study was the interchange of knowledge, which was reciprocal between attendees and HCPs, between attendees themselves, and between attendees and their whānau/families or communities. Knowledge itself could be considered a relationship; it required reciprocity and trust to “bring it alive”, and not as a one-way exchange of the HCP, from a position of “power” telling the “patient” what to do. This knowledge empowerment could be likened to social capital—a “knowledge capital”—that can only enhance “societies”-management of T2D, beyond that of “self”-management. Allory et al. (43) stress that a key objective of T2D education programmes should be to develop new social links for attendees as social difficulties and concerns can create limited social access and promote isolation for marginalised populations. We suggest that growing both social and knowledge capital should be key aims of lifestyle programmes such as DCEP and primary care providers should be supported centrally (by government health policies and funding) to support such initiatives.

The strength of this study is in the rich and diverse data collected. Limitations include the potential standpoint of participants, in that DCEP may have attracted both people wishing to be involved in a community lifestyle programme and HCPs who were sympathetic with the ethos of the programme and who already held a positive belief of its benefits. Further, these interviews occurred post the initial twelve-week programme and thus did not capture participant's perspectives of the maintenance ongoing part of DCEP. Whilst we had ethnic diversity in our participants and interviewers, this diversity was not extended to the research team analysing the data, thus preventing a Māori perspective being applied to the analysis.

We explored what aspects of DCEP were considered by both attendees and HCPs to be important to its person/whānau-centeredness approach. It is ‘all about relationships’ was the key aspect—all other factors related back to the development and maintenance of relationships. These relationships were between all whānau involved—the attendees, their family, the wider community, the healthcare provider organisations, and the HCPs and personnel involved in delivering DCEPs. These relationships created an accepting, understanding and social atmosphere that enabled attendance and facilitated both knowledge exchange and ability to exercise, both considered to be beneficial by attendees. There is nothing novel about us purporting to deliver DCEP in a person-centred care approach, indeed such an approach to the management of T2D is promoted by agencies such as the NZ Ministry of Health (1) and the American Diabetes Association (49). How a “person-centred care approach” is interpreted however and thus delivered can differ from an insincere and superficial application (46) to one that embraces the models such as those described by McCormack (22) and McKenzie et al. (37). As the latter are described within a nursing model of healthcare, understanding the nuances of such an approach within the interprofessional rehabilitation context of T2D may enable a more considered application. The nature and role of person-centred care in the management of long-term health conditions is complex, and in the context of rehabilitation and long-term health conditions, the concept is still evolving (18–20). The key rehabilitation implication from our study is that taking time to develop relationships with all involved, not just the patient, is important and worthwhile and that “person-centred care” should perhaps evolved to be “whānau-centred care”.

## Data Availability Statement

The raw data supporting the conclusions of this article will be made available by the authors, without undue reservation.

## Ethics Statement

The studies involving human participants were reviewed and approved by New Zealand Health and Disability Ethics Committee, Ministry of Health (HDEC17/CEN/241/AM01). The patients/participants provided their written informed consent to participate in this study.

## Author Contributions

LH was the principle investigator of the research project and wrote the introduction, methods and discussion sections of the paper and edited the findings sections. CH was the lead clinical author on this paper and organized and supervised all the clinical staff involved in the study, developed DCEP, and contributed significantly to data analysis and editing of the paper. DK was the lead project manager and organized all the interviews and facilitated a number of the interviews and contributed significantly to data analysis and editing of the paper. CS led and did most of the qualitative analysis of this project and led the writing of the findings section of the paper and assisted editing the paper overall. All authors contributed to the article and approved the submitted version.

## Funding

This study is funded by the New Zealand Health Research Council (HRC Project Grant 17/233).

## Conflict of Interest

The authors declare that the research was conducted in the absence of any commercial or financial relationships that could be construed as a potential conflict of interest.

## Publisher's Note

All claims expressed in this article are solely those of the authors and do not necessarily represent those of their affiliated organizations, or those of the publisher, the editors and the reviewers. Any product that may be evaluated in this article, or claim that may be made by its manufacturer, is not guaranteed or endorsed by the publisher.
